# Near-Infrared Spectroscopy-Based Phenomics Data Can Improve Genomic Prediction of Agronomic and Grain Quality Traits Across Multi-Environment Sorghum Hybrid Trials

**DOI:** 10.3390/plants14182871

**Published:** 2025-09-15

**Authors:** Pradip Sapkota, Jales Fonseca, Ramasamy Perumal, José Crossa, William L. Rooney

**Affiliations:** 1 Department of Soil and Crop Sciences, Texas A&M University, College Station, TX 77843, USA; 2 Research and Development Breeding, Bayer Crop Science-US, Stanton, MN 55018, USA; 3 Agriculture Research Center, Kansas State University, Hays, KS 67601, USA; 4 Maize and Wheat Improvement Center (CIMMYT), Texcoco de Mora 56237, Mexico

**Keywords:** genomic selection, grain quality, near-infrared spectroscopy, phenomic selection, sorghum

## Abstract

In recent years, phenotyping approaches in plant breeding have expanded in both methodology and data collection capacity. One such tool, Near-Infrared Spectroscopy (NIRS) generates a wealth of reflectance values for biological samples. To test the potential of NIRS-based predictions, a hundred grain sorghum hybrids generated from a 10 × 10 factorial mating design were evaluated across eight environments. Hybrids were phenotyped for grain yield, days to anthesis, plant height, kernel hardness index, kernel diameter, and kernel weight. Hybrid grain samples were scanned with NIRS to generate phenomic data while parental lines were genotyped using genotyping by sequencing. Three different predictive models: genomic prediction (GP), phenomic prediction (PP), and GP + PP were fitted. Three different cross-validation schemes of untested hybrids in characterized environments (CV1), tested hybrids in uncharacterized environments (CV2), and untested hybrids in uncharacterized environments (CV3) were completed. GP + PP significantly improved over GP for days to anthesis, kernel hardness index, kernel diameter, and kernel weight for CV1. Prediction accuracy of GP + PP was also significantly improved for the kernel hardness index and kernel weight for CV2 and CV3. Depending on logistics, phenomic prediction has the potential to complement or supplement genomic data for predictive strategies in sorghum.

## 1. Introduction

Classical crop breeding strategies have integrated new prediction methodologies to meet increased productivity demands for food, feed, fiber, and forage [1]. Of these, genomic selection (GS) and novel approaches to phenomics are among the most important new technologies [2]. While these technologies have the greatest impact on the most widely grown and marketed crops, other crops such as sorghum [*Sorghum bicolor* (L.) Moench] could benefit from their use as well. Grain sorghum is an internationally grown grain crop that is used as a food, feed, and industrial purpose [3]. While it has always been a food crop in Africa and India, food-grade sorghum has increased in other cultures due to its low glycemic index and gluten-free properties [4,5].

While improvements in sorghum genetics and agronomic management have increased grain yield, the rate of genetic improvement has slowed in recent years [6,7]. The adoption of newer breeding technologies could improve the rate of genetic gain. To date, a primary limitation to their use in sorghum is the absence of the logistical infrastructure [8].

One of these approaches is GS, which trains a statistical model by integrating phenotypic data and genome-wide molecular markers to predict the performance of untested cultivars [9]. GS has accelerated rates of genetic gain and fits well within most breeding programs [10]. Another approach, high-throughput phenotyping, can measure traits faster and more efficiently than the traditional approaches [11]. One such method, near-infrared spectroscopy (NIRS), is commonly used to predict grain compositional traits without extensive sample preparation and destruction. NIRS-based phenomic data can be collected at a lower cost and easily as compared to genomic data. Predictions are based on reflectance values from different wavelengths that are associated with targeted organic compounds [12,13].

In addition to direct prediction of a specific trait, raw NIRS band reflectance values could also be used for more general predictive modeling purposes [14]. Performing selection for targeted traits based on large numbers of reflectance as variables through predictive modeling is known as phenomic selection (PS) [14,15]. Although the cost of genotyping has dropped, implementation of GS can be challenging for an exponentially larger number of genotypes [16]. In addition, PS is logistically less demanding in comparison to GS; therefore, smaller breeding programs as well as crops with limited genomic resources can successfully adopt it [14]. Combined or independently, GS and PS have the potential to improve selection intensity, accuracy, and ultimately genetic gain improvement in a breeding program, and the successful implementation of these tools has been demonstrated in corn, wheat, and soybean [17,18].

Earlier work in sorghum demonstrated that genomic prediction could estimate breeding values for parental lines and the performance of specific hybrid performances for traits such as grain yield (GY), plant height (PH), and days to anthesis (DA) [19,20,21,22]. Given the increased use of sorghum as a food grain, there is increased interest in grain quality improvement in sorghum [23]. Of those, kernel physical traits such as the kernel hardness index (KHI), kernel diameter (KD), and kernel weight (KW) are typically characterized at the level of a single kernel with a single-kernel characterization system (SKCS), manufactured by Perten Instruments AB. KHI represents end-use quality traits such as mold resistance, grain storage ability, and insect resistance [24,25]. KD and KW, which represent grain size and kernel weight, are also directly relevant to end uses dictating human consumption [26]. However, these SKCS instruments are no longer available for commercial purposes, creating a void for phenotyping. To accelerate grain quality improvement in sorghum, alternative methods of phenotyping for these traits must be developed. In this context, widespread adoption of NIRS within breeding programs provides an opportunity to optimize NIR-based phenomic selection approaches to accelerate grain quality improvement in sorghum.

To test the potential of integrating GS and PS in sorghum, a set of 100 sorghum hybrids generated from a 10 × 10 factorial mating design were evaluated across eight environments in Texas and Kansas dryland locations. The hypothesis of this study is that NIR reflectance data obtained on grain samples can serve as low-cost phenomic data, alone or in combination with genomic data, and could accurately predict agronomic performance and grain quality traits of hybrid sorghums. The objectives of the study were to (1) estimate variance components and heritability for NIRS values by band reflection, (2) build prediction models for agronomic and kernel traits in a single environment, and (3) build multi-environment prediction models by integrating genomic and phenomic data across environments for different prediction scenarios.

## 2. Results

### 2.1. Combined Analysis and Variance Components

**Figure 1:**
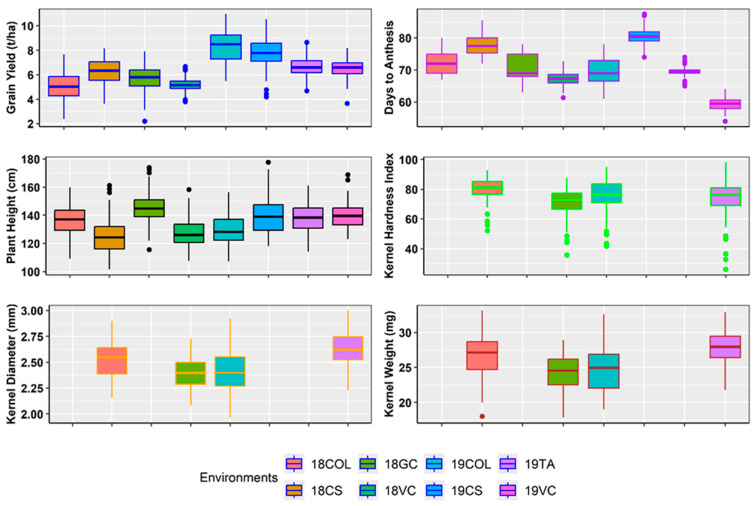
Distribution of best linear unbiased estimates of grain yield (t ha^−1^), days to anthesis, and plant height (cm) for hybrids evaluated across eight environments: 18COL (2018 Colby, KS, USA), 18CS (2018 College Station, TX, USA), 18GC (2018 Garden City, KS, USA), 18VC (2018 Victoria, TX, USA), 19COL (2019 Colby, KS, USA), 19CS (2019 College Station, TX, USA), 19TA (2019 Taft, TX, USA), and 19VC (2019 Victoria, TX, USA); best linear unbiased estimates of kernel physical factors: kernel hardness index, kernel diameter, and kernel weight for four representative environments: 18CS (2018 College Station, TX, USA), 18VC (2018 Victoria, TX, USA), 19COL (2019 Colby, KS, USA), and 19VC (2019 Victoria, TX, USA).

Significant variation was observed for GY, DA, PH, KHI, KD, and KW among hybrids within all environments. The average best linear unbiased estimate (BLUE) for grain yield combined across eight environments was 6.44 t ha^−1^ with a range from 2.2 to 10.98 t ha^−1^. The average BLUE across eight environments for DA varied from 54 to 87.5 days, with a mean of 70.8 days, and PH varied from 101.6 to 177.8 cm with an average of 135.4 cm. For kernel physical characteristics, BLUE of KHI ranged from 26.1 to 98.3 with a mean of 75.2, KD ranged from 1.96 to 3.002 mm with a mean of 2.49 mm, and KW ranged from 17.82 to 33.19 mg with a mean of 25.87 mg (Figure 1).

The likelihood ratio test (LRT) for combined analysis across environments (using Equation (3)) revealed significant effects of hybrids, environment, and interaction of GCA and SCA effects with environments for GY, DA, PH, KHI, KD, and KW (Table 1). G × E interactions for GY, DA, and PH ranged from 4.9 to 12.1; the same for KHI, KD, and KW were slightly higher (10.8–19.4) (Table 1). Environmental effects accounted for 49%, 78%, and 24% of the variation for GY, DA, and PH, respectively. Likewise, hybrid effects accounted for 9%, 12%, and 47% of the variation for GY, DA, and PH, respectively. In general, kernel traits had larger hybrid effects than environmental effects; hybrid effects accounted for 57%, 54%, and 42% of KHI, KD, and KW, respectively. In terms of specific genetic effects, the additive effect was larger in males than in females for all three kernel traits (Table 1). The G × E interactions were significant for both agronomic and kernel traits but lower than the main effects for both hybrids and environments. Broad-sense heritability (H^2^) was high for all traits, and narrow-sense heritability (h^2^) was only slightly lower, indicating that additive gene action predominates for these traits (Table 1). The h^2^ estimates for male and female parents were similar for GY and DA, but h^2^ estimates for the male parent were larger than those of female parents for PH, KHI, KD, and KW (Table 1).

### 2.2. Variance Components and Heritability of NIR Spectra

**Figure 2:**
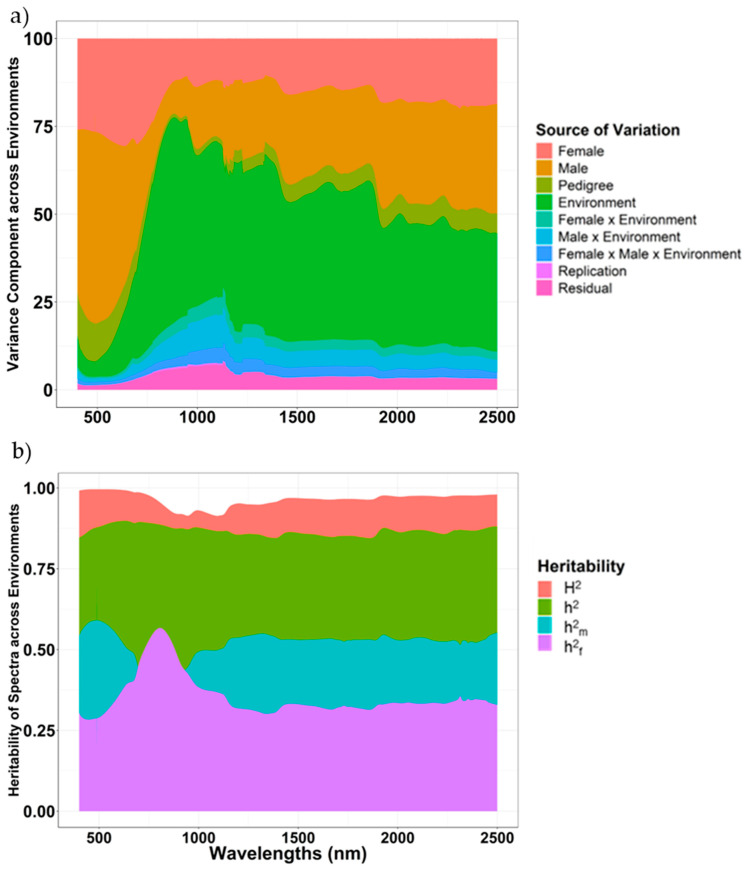
(**a**) Stacked graph presenting percent variation explained by female effects, male effects, female × male effects, environment effects, female × environment effects, male × environment effects, and replication effects across spectral bands of 400 nm to 2500 nm across eight environments by Equation (3), where $y=1µ+Z_{1}f+Z_{2}m+Z_{3}h+Z_{4}E+Z_{5}fE+Z_{6}mE+Z_{7}fmE+Z_{8}r\;(E)+\;e$. (**b**) Overlaying graphs presenting broad sense heritability (H^2^), total narrow sense heritability (h^2^), female NSH (h^2^_f_), and male NSH (h^2^_m_) for spectral bands of 400 nm to 2500 nm across environments.

**Figure 3:**
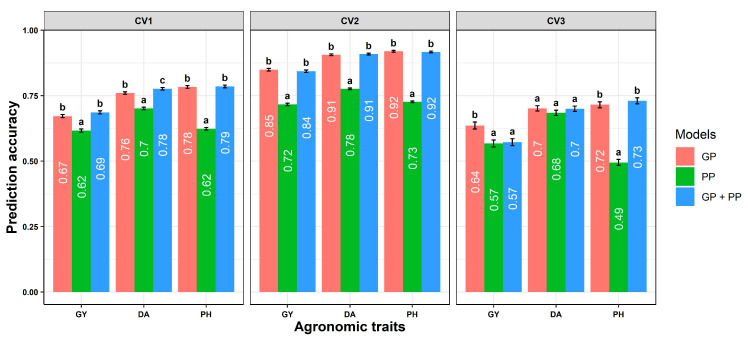
Prediction ability of genomic prediction (GP), phenomic prediction (PP), and GP + PP models for agronomic traits grain yield (GY), days to anthesis (DA), and plant height (PH) in three prediction scenarios: CV1 (untested hybrids in a characterized environment), CV2 (tested hybrids in an uncharacterized environment), and CV3 (untested hybrids in an uncharacterized environment) across environments. Error bars and significant letters a, b, and c were assigned for statistical differences determined by Tukey’s honest test between models.

In the combined analysis, using Equation (3) for all 4200 reflectance bands, female effects explained an overall of 17.6% of the variation, ranging from 1.42 to 30.6%. Male effects explained 26.3% of the variation with a range of 8.91 to 64.3% (Figure 2a). Female × male effects ranged from 1.1 to 12.7% of the variation with an average of 5.1%. Combined, the total hybrid variation (female + male + female × male) accounted for 49%, which indicates that spectra can delineate genotypic differences across environments. Environment variation was 37.1%, ranging from 3.75 to 58.3% (Figure 3). G × E effects explained 2.1% of total variation at spectrum of 487.5 nm, whereas about 20.8% of the variation was explained at spectrum of 1127.5 nm with a mean of about 9.99% (Figure 2a). Majority of variations were attributed to genetic effects with prominent male effects when analyzed within environments (Supplementary Figure S1). H^2^ for reflectance values of spectra ranged from 0.91 (1098.5 nm) to 0.99 (487.5 nm). Most of these variations were driven by additive effects, as the h^2^ range was from 0.85 (1857 nm) to 0.90 (630 nm). Further partitioning revealed that female h^2^ ranged from 0.21 (487.5 nm) to 0.57 (805.5 nm); however, male h^2^ ranged from 0.32 (807 nm) to 0.69 (487.5 nm) (Figure 2b).

### 2.3. Predictive Abilities of Genomic or Phenomic Prediction Models

The prediction accuracy significantly varied across traits and models. In the combined analysis, GP consistently had a higher prediction accuracy in CV1 than PP for agronomic traits in hybrids (Figure 3). While GP + PP numerically outperformed GP in the scenario of CV1, it significantly outperformed GP for DA (Figure 3). Likewise, PP statistically performed similarly to GP for DA in CV3 (Figure 3).

**Figure 4:**
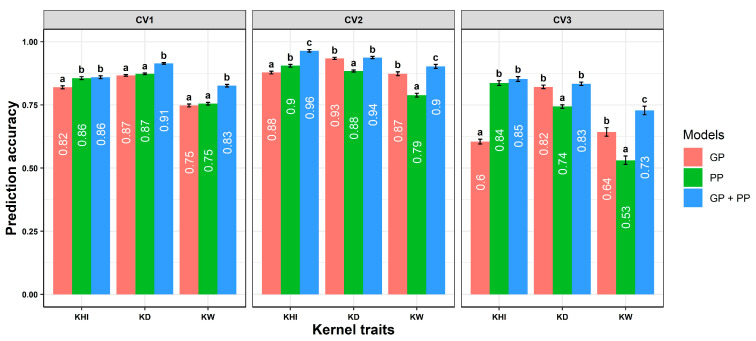
Prediction ability of genomic prediction (GP), phenomic prediction (PP), and GP + PP models for kernel physical characteristics: kernel hardness index (KHI), kernel diameter (KD), and kernel weight (KW) in three different prediction scenarios: CV1 (untested hybrids in a characterized environment), CV2 (tested hybrids in an uncharacterized environment), and CV3 (untested hybrids in an uncharacterized environment) across environments. Error bars and significant letters a, b, and c were assigned for statistical differences determined by Tukey’s honest test between models.

For kernel traits, PP numerically outperformed GP in the CV1 scenario, and prediction was significantly improved for the GP + PP combined model (Figure 4). In the CV2 and CV3 prediction scenarios, GP was less effective than PP or GP + PP for KHI and KW; however, GP was more effective than PP for KD; and there was significant improvement in the GP + PP model for KHI and KW.

### 2.4. Relationship of NIRS Bands with Phenotypic Traits

**Figure 5:**
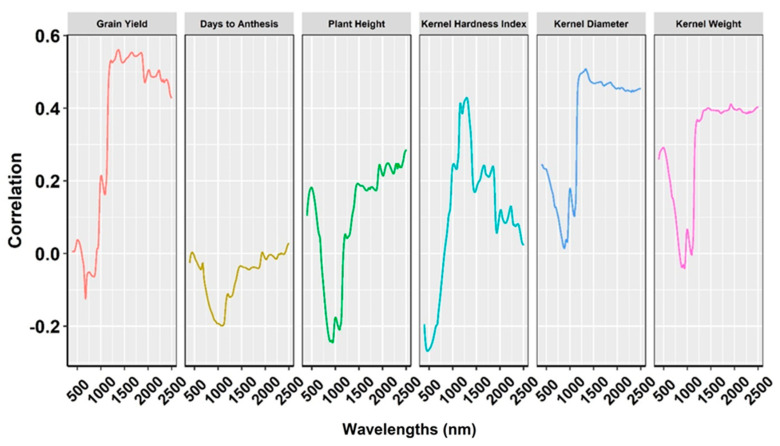
Pearson’s correlation of NIR bands with grain yield, days to anthesis, plant height, kernel hardness index, kernel diameter, and kernel weight across spectral wavelengths of 400 nm–2500 nm across environments.

Correlations between all six traits and the specific bandwidth varied (Figure 5). For example, correlations between bandwidths and GY were highest (r = 0.56) at 1370 nm, while KHI correlations peaked (r = 0.46) at 1297 nm. Other peak correlations were r = 0.51 and 0.46 for KD (1331 nm) and KW (1923 nm), respectively. All the traits with higher prediction accuracy had their highest correlation with bands from 1200 nm to 1400 nm, which is the short-wavelength infrared region. Both DA and PH had no functional relationship with bandwidth at any wavelength (Figure 5). Interestingly, these traits had comparable prediction accuracies with phenomic and genomic prediction models.

## 3. Discussion

Previous studies have reported the inheritance and the genetic variances of GY, PH, and DA [19,21], but the inheritance and the genetic variances of kernel traits are unique and represent some insight into the quantitative nature of these traits. Significant variation was detected among the hybrids, and the traits had high heritability for all three kernel traits (Table 1). Variation was detected in both the male and female parents, but the male parents accounted for a higher proportion of additive variation. Interestingly, the SCA effects for kernel traits were high relative to the agronomic traits, and this implies the influence of dominant gene effects for these traits. The largest variation for each of the three agronomic traits was due to the environmental effect, whereas genetic and G × E effects were the primary sources of variation for kernel traits. Given that selection for these traits is, at best, indirect, implies that it could shift means quickly towards any directional selection, although the significant G × E interactions for these traits must be considered in selection [27].

Earlier studies highlighted remote sensing as a useful phenomic selection tool for assessing plant health and grain yield in sorghum [28]. Motivated by studies in other crops such as corn, wheat, and coffee, this study represents the first report of NIR-based phenomic prediction methods for agronomic performance and grain quality traits in sorghum [29,30,31]. As breeding programs have started implementing the GS approach, optimization of GS models is necessary to maintain consistent prediction accuracy, which could be achieved with the inclusion of other omics data [30,32,33]. NIR reflectance information, as low-cost omics data, has proven effective in complementing or supplementing genomic data for predictive modeling approaches [29,34]. This study explored the potential of implementing NIR bands alone or combining them with genomic data to train predictive models in sorghum hybrids.

NIR-based phenomic data appear useful in predicting economically important traits in sorghum. Using variance estimations from NIR bands, it was possible to consistently detect genetic, environmental, and G × E interaction effects across different bands (Figure 2a). The majority of variation in NIR spectra was attributable to additive genetic effects (female and male), highlighting the potential for genetic improvement through phenomic selection strategies [31]. Variations in NIR bands have been dissected in a similar manner in other crops as well [14,35,36]. The highly heritable nature of NIR spectra, along with major variations attributable to additive effects, demonstrates replicability for their regular use in a breeding program (Figure 2b). Overall, genetic variation in spectra was primarily attributed to male effects (Supplementary Figure S1), revealing that NIR spectra could be used to distinguish between hybrids in a manner akin to molecular markers. It should be noted that grain quality traits had larger genetic effects and more accurate predictions than agronomic traits. However, they were evaluated in fewer environments, which may have subjected these traits to lower G × E interaction effects. Consequently, higher prediction accuracies of phenomic models for three grain quality traits could be due to efficient discernment of NIR bands among grain samples. As expected, the relative effect of any bandwidth varied, and some were more correlated with genetics, while specific bandwidths were affected by the environment.

For untested hybrids in characterized environments (CV1), GP resulted in higher prediction accuracy than PP for agronomic traits, and prediction accuracy slightly increased (approximately 1.5%) for GP + PP, except for plant height (Figure 4). Similar results were reported in wheat [35,37]. The higher prediction accuracy observed in the GP + PP model may be due to the ability of NIR to consistently differentiate the hybrids. While the relationship between NIR reflectance and maturity is not obvious, the results indicated that NIR captured variation unexplained by GP, given the improvement in prediction accuracy for GP + PP. NIR can capture non-additive effects more consistently [35]. However, this is not always true; sorghum plant height is strongly influenced by non-additive gene action, but prediction accuracy did not improve with NIR band reflectance data. Thus, the typical improvements seen with NIR-based prediction models likely depend on the relationship between the trait and the physiochemical properties of the grain.

GP outperformed PP for GY, DA, and PH for untested hybrids in characterized environments (CV2), and GP + PP did not improve prediction accuracy. For untested hybrids in uncharacterized environments (CV3), GP outperformed PP for GY and PH, while PP was statistically similar to GP for DA. Similarly, within-environment predictability of GP was consistently better than that of PP, with additional gains observed when combining GP + PP for agronomic traits (Supplementary Figure S2).

In the CV1 scenario, PP statistically outperformed GP for KHI, and further significant improvements were observed in the GP + PP for KD and KW. Notably, PP significantly outperformed GP for KHI by approximately 24% in CV3 (Figure 4). Collecting grain samples from new environments can accurately predict KHI via phenomic models as compared to genomic models. The combined model GP + PP also improved prediction accuracy for KW by about 4% in CV2 and 9% in CV3. The high prediction accuracy of PP relative to GP in CV2 and CV3 indicated its potential for predicting hybrids in uncharacterized environments. Similar patterns of improvement were recorded for GP + PP over GP for most of the single-environment models (Supplementary Figure S2). The superior performance of PP in predicting grain quality traits stems from their association with the physical structure of grain (Figure 4) [38]. Higher correlation was observed for most of the traits with respective NIR bands, except for plant height and days to anthesis (Figure 5). Grain yield and kernel weight were highly correlated with spectral bands in corn [30]. Inclusion of LASSO-nominated significant bands in corn recorded no improvement in prediction accuracy; therefore, fitting all the available bands would be effective for predictive modeling [30].

The whole-kernel NIR is effective at predicting hardness in wheat [39] and results herein indicate the same is true of sorghum. Thus, with further testing, NIR could offer a non-destructive alternative to the SKCS method. This could provide alternative methods for time-consuming and destructive SKCS methods that are rapidly disappearing from grain quality labs. Phenomic selection models can utilize the same single spectral scan to predict complex traits; therefore, it is rapid and less time-consuming. While these methodologies can be extended to other crops and traits, the efficacy of phenomic models is largely subject to the relationship between grain structure and traits of interest. For instance, phenomic models can be implemented to improve traits such as amylose, amylopectin, lysine and 3-DOA (3-deoxyanthocyanidins), etc., in suitable germplasm, specialty sorghum such as black sorghum and waxy sorghum, to accelerate trait-based breeding efforts.

The effectiveness of phenomic prediction in sorghum varies depending on the trait, with the greatest success observed for traits that are closely associated with the chemical composition of grain. This is a logical extension as NIRS detects chemical bonds that determine tissue composition, which are in turn influenced by both genetic and environmental factors [35]. NIRS effectively captures and tags specific regions of the reflectance spectrum, analogous to how QTL are identified in genomics [40]. As such, NIR reflectance data may also have the potential to consistently reveal genetic architecture underlying certain traits.

The positive correlation between phenomic and genomic predictions across traits indicated that spectral data exhibits a level of consistency comparable to that of genetic markers and phenotypic data [41]. Unlike genomic prediction, phenomic prediction is less dependent on the size of training sets and genetic relationships between individual hybrids and more influenced by G × E interactions [42]. Additionally, phenomic prediction is less prone to overestimation due to relatedness between training and testing sets, which can affect the accuracy of genomic prediction. The integration of high-dimensional phenomic datasets that capture underlying sources of variations has shown potential for enhancing prediction accuracy in this study and in others [43].

Although phenomic-assisted breeding has demonstrated efficiency, it remains less commonly used than genomic-assisted breeding in cultivar development pipelines [44]. Limited application of PS might have been associated with the challenges associated with cost, labor, time, and processing [16]. One of the major caveats of grain-based omics data, as compared to genomic data, is the requirement for multi-location trials to capture spectral data across diverse environments. In addition, phenomic data are influenced by environmental factors and G × E interactions, which can obscure reliable genetic differences among lines [42]. However, the high repeatability of NIR spectra observed in this study indicates that fewer environments may be sufficient for reliable data collection.

This study acknowledges the disadvantage of PP over GP, as there is still a need for hybrid development, grain sampling, and NIR scanning, while GP can be implemented by using molecular markers obtained from parents. GP models could be effective for breeding programs that already have logistics developed, while PP can be useful to the breeding programs that are at a disadvantage in genotyping. Smaller breeding programs, having limited genomic resources, for example, in developing countries, e.g., Asian and African countries, can take advantage of this method. While GP demands integration of genotyping platforms, maintaining relatedness, and appropriate training size; PP is less influenced in such scenarios. Implementation of PP into a breeding program does not require extensive redesigning in terms of logistics. While deciding on the efficacy of PP, it is usually benchmarked against GP. Prediction accuracy was typically considered as a metric to show that a model is better than others. However, more accurate models do not necessarily have better prediction accuracy, and relying solely on prediction accuracy is not sufficient. The application of prediction models impacts other parameters of the breeder’s equation and the rate of genetic gain must be estimated by considering the full effects of their application [45].

In a sorghum improvement program employing GP, a subset of lines, such as doubled haploids and lines from the F_4_ generation, are testcrossed to generate hybrids for advanced testing and to serve as training sets. The success of GP in such a system depends on maintaining consistent prediction accuracy across cycles. Integrating the phenomics data, specifically the NIR spectra obtained from grain samples collected during hybrid trials, could enhance the prediction accuracy of GP models. Notably, since mechanical harvesting systems often include built-in NIR prediction capabilities, these spectral data are already available and can be utilized without incurring additional resource demands.

Multi-environment breeding trials are often subject to biotic and abiotic stresses, which can limit the collection of reliable phenotypic data. In such cases, collecting grain samples from selected environments may reduce the need for extensive phenotyping, as NIRS has the potential to capture genotype, environment, and G × E interaction effects [46]. PP models can be trained to predict hybrid performance across environments by leveraging spectral similarities between both hybrids and environments [47]. This could improve the phenotypic data availability to train GP models to improve breeding outcomes [45]. These methodologies could be further enhanced by using spectral data collected at early growth stages such as from vegetative tissues using a hand-held spectrophotometer rather than waiting until grain maturity. Training PP models with early growth stage spectra may improve practical applications in the breeding program. Improvement in prediction accuracy in multi-trait genomic selection models for grain quality traits, suggests a direction for similar advancements in phenomic selection [48]. Consequently, multi-trait phenomic prediction models should be explored further in sorghum.

## 4. Conclusions

This study investigated the potential of NIR-based phenomic prediction models to increase the selection efficacy of hybrids from applied sorghum breeding programs. This first-time study indicates that NIR-based phenomic prediction can be effective for certain traits in sorghum. Specifically, phenomic predictions were comparable to or exceeded genomic predictions for grain yield, days to anthesis, kernel hardness index, kernel diameter, and kernel weight. However, phenomic prediction was notably less effective for plant height. A consistent improvement in prediction accuracy was observed when phenomic data were integrated into genomic prediction models. These results suggest that genomic prediction remains preferable for traits not closely associated with grain characteristics, whereas phenomic data can add value when relevant samples are available. Nonetheless, the utility of phenomic prediction is limited by the prerequisite of obtaining phenotypic samples, which may not align with early-stage genomic selection strategies that precede phenotyping. Therefore, phenomic prediction could serve as a complementary tool to genomic prediction, particularly for materials lacking genotypic data or in later stages of selection. Future research studies should explore the integration of phenomic data derived from remote sensing technologies and include more genetically diverse materials across a broader range of environments. In this study, smoothing phenomic data using first derivatives produced results like genomic prediction models. Advancing this work will require more robust machine learning models capable of integrating high-dimensional data to improve the prediction of economically important traits in sorghum.

## 5. Materials and Methods

### 5.1. Experimental Design

A total of 100 hybrids were developed using a 10 × 10 complete factorial mating design. These hybrids were grown in eight different environments in summer 2018 and 2019. Details on hybrid development and field evaluation were described in a previous publication [19]. In brief, the experimental design in each environment was a randomized complete block design (RCBD) with three replications in 2018 and two replications in 2019. The experiments in 2018 were conducted in four environments: Victoria, Texas: 18VC (28°47′24.4″ N, 96°50′22.6″ W), College Station, Texas: 18CS (30°32′56.6″ N, 96°26′11.5″ W), Garden City, Kansas: 18GC (37°59′21.4″ N, 100°48′52.5″ W), Colby, Kansas: 18COL (39°22′56.6″ N, 101°04′45.0″ W). In 2019, the same experiments were conducted in Victoria, College Station and Taft (19TA, 28°00′05.4″ N, 97°15′12.4″ W), Texas, and Colby, Kansas.

### 5.2. Agronomic Evaluation and Traits

Data on three agronomic traits: days to anthesis (DA), plant height (PH), and grain yield (GY) were recorded on a plot basis uniformly in all the locations studied. The DA was the number of days from planting to when half of the plot flowered halfway down the panicle. For PH, a representative plant from a plot was measured from the ground to the tip of the panicle. At physiological maturity, the crop was harvested using a plot combine; grain was weighed, and moisture content was measured. Plot weights were later adjusted for 14% of moisture to calculate GY on a ton/hectare (t/ha).

### 5.3. Grain Characterization

A grain sample, collected on a plot basis, and composed of five panicles typical of the plot was hand-harvested prior to combine harvest. The panicles were threshed in Almaco BT14E Belt Thresher (ALMACO), and grain samples were dried to a shelf-stable moisture content averaging 12%. A 100 g grain sample from each hybrid in each environment was scanned using a FOSS 2500 spectrophotometer (FOSS North America, Eden Prairie, MN, USA, 2005), which measures reflectance between 400 and 2500 nm at an interval of 0.5 nm. By using the calibrated curves available in the Texas A&M sorghum breeding and genetics laboratory, these spectra were estimated for starch, protein, fiber, fat, ash, and moisture content in the grain (on a % basis). In addition, 300 grains of each plot from four representative environments (18CS, 18VC, 19COL, and 19VC) were analyzed for the three physical traits such as the kernel hardness index (KHI), kernel diameter (KD), and kernel weight (KW), using the single-kernel characterization system (SKCS 4100, Perten Instruments North America Inc., Springfield, IL, USA). The value for KHI was presented in numbers ranging from 0 to 100, where a higher number indicates a harder kernel.

### 5.4. Phenomic Data

The raw NIR spectra generated to estimate grain composition were also used to calculate the best linear unbiased estimates (BLUEs) for each hybrid within environments for all wavelengths by solving mixed models presented in Equation (1). ‘y’ represents the vector of the response variable, h represents the hybrid fixed effect, r represents replications, and e represents residuals in Equation (1). Likewise, *Z*_1_ and *Z*_2_ are incidence matrices relating phenotypes with hybrids and replications, respectively. The BLUEs of phenomic data have dimensions of 100 × 4200 and 800 × 4200 within and across all environments, respectively. Data smoothing and pretreatment with the first derivative were performed using the ‘SavitzkyGolay’ function with a window of 11 to remove low-quality spectra. Filtered data were scaled around zero to generate even distribution. The dimensions of phenomic data were reduced to 100 × 4190, and 800 × 4190, respectively, for single and all environments. All pretreatments were performed using the package ‘prospectr’ in R 4.3.1 [49]. These clean data were used to build the relationship among hybrids. Phenomic relationship matrices were computed as $\frac{NIR{\times NIR}^{’}}{4190}$, where NIR was the scaled first derivative of phenomic data, and NIR’ was the transpose of NIR.

(1) $$y=1µ+Z_{1}h+Z_{2}r+e$$

### 5.5. Genotypic Data

DNA extraction, quality control, and library preparation were performed to perform genotyping by sequencing (GBS) for parents used in this study [50]. All the steps for mapping and variant calling were detailed in a previous publication [19]. Single Nucleotide Polymorphic (SNP) markers with more than 5% missing values were removed and missing values were imputed. Finally, 35,546 SNP markers were used to build a genomic relationship matrix, where alleles were coded as 0, 1, 2; 0 = homozygous recessive, 1 = heterozygote, and 2 = homozygous dominant. A genomic relationship matrix was calculated using the ‘vanraden’ method in R [51]. In the genomic relationship matrix among parents, G would be G = (XX’)/n, where X is the SNP marker of parents centered around zero, X’ is the transpose of X, and n is the number of 35,546 SNP markers used. These relationships were used to build kernels to model genetic effects via general combining abilities (GCAs) and specific combining abilities (SCAs) to fit into predictive models.

### 5.6. Variance Component Estimations

Phenotypic data and reflectance values were fitted in a linear mixed model, and the effects of female and male parents, hybrid (female × male) effects, and replications within environment were estimated using Equation (2).

(2) $$y=1\mu +Z_{1}f+Z_{2}m+Z_{3}h+Z_{4}r+e$$

where y is a vector of response variables; µ is an intercept; f is a random effect of female, f ~ N (0, σ^2^_f_I); m is a random effect of male, m ~ N (0, σ^2^_m_I); h is a random effect of hybrid h ~ (0, σ^2^_h_I); r is a vector of replications r ~ N (0, σ^2^_r_I); e is a vector of residuals, e ~ N (0, σ^2^_e_I); 1 is a vector of ones; ***Z_1_***, **Z_2_**, **Z_3_**, and ***Z_4_*** are incidence matrices; σ^2^_f_, σ^2^_m_, σ^2^_r,_ and σ^2^_e_ are variance components of females, males, hybrids, replicates and residuals, respectively. For combined-environment analysis, Equation (2) was extended to include environment and genotype × environment (G × E) and performed the following combined analysis:

(3) $$y=1\mu +\;Z_{1}f+Z_{2}m+Z_{3}h+Z_{4}s+Z_{5}fs+Z_{6}ms+Z_{7}fms+Z_{8}r(s)+\;e$$

For combined analysis, s is a vector of environmental effects, s ~ N (0, σ^2^_s_I); fs is a vector of the interaction effect between the GCA of female and environmental effects, fs ~ N (0, σ^2^_fs_I); ms is a vector of the interaction effect between the GCA of male and environmental effects, ms ~ N (0, σ^2^_ms_I); fms is a vector of the interaction effect between the SCA of hybrid combinations and environmental effects, fms ~ N (0, σ^2^_fms_I); r(s) is the vector of replication nested within environments, r(s) ~ N (0, σ^2^_r(s)_I). Variance components for response variables were estimated via restricted maximum likelihood (REML), and significance was assessed by the likelihood ratio test (LRT) at a 5% level. All analyses were performed on the lme4 package in R. Based on variance component estimations, broad sense heritability and narrow sense heritability were computed for a single environment: $H^{2}=\;\frac{\sigma_{g}^{2}}{\sigma_{g}^{2}+\;\frac{\sigma_{e}^{2}}{r}}$ and $h^{2}=\;\frac{\sigma_{a}^{2}}{\sigma_{h}^{2}+\;\frac{\sigma_{e}^{2}}{r}}$. where $\sigma_{g}^{2}$ is the variance component due to hybrids, $\sigma_{a}^{2}$ is additive genetic variance, $\sigma_{e}^{2}$ is error variance, and r is number of replications in each experiment. Narrow sense heritability for females ($h_{f}^{2}$) and males ($h_{m}^{2}$) within an environment was computed: ${h_{f}}^{2}=\;\frac{\sigma_{f}^{2}}{\sigma_{g}^{2}+\;\frac{\sigma_{e}^{2}}{r}}$ and ${h_{m}}^{2}=\;\frac{\sigma_{m}^{2}}{\sigma_{g}^{2}+\;\frac{\sigma_{e}^{2}}{r}}$, respectively. These equations were extended for combined analysis: $H^{2}=\;\frac{{\sigma_{g}^{2}}^{*}}{{\sigma_{g}^{2}}^{*}+\;\frac{{\sigma_{gs}^{2}}^{*}}{t}+\frac{\sigma_{e}^{2}}{tr}\;}$, $h^{2}=\;\frac{{\sigma_{a}^{2}}^{*}}{{\sigma_{g}^{2}}^{*}+\;\frac{{\sigma_{gs}^{2}}^{*}}{t}+\frac{\sigma_{e}^{2}}{tr}\;}$, ${h_{f}}^{2}=\;\frac{\sigma_{f}^{2}}{{\sigma_{g}^{2}}^{*}+\;\frac{{\sigma_{gs}^{2}}^{*}}{t}+\frac{\sigma_{e}^{2}}{tr}\;}$, and ${h_{m}}^{2}=\;\frac{\sigma_{m}^{2}}{{\sigma_{g}^{2}}^{*}+\;\frac{{\sigma_{gs}^{2}}^{*}}{t}+\frac{\sigma_{e}^{2}}{tr}\;}$ where ${\sigma_{g}^{2}}^{*}$ is the variance component due to hybrids that were computed as the summation of the variance component due to female ($\sigma_{f}^{2}$), male ($\sigma_{m}^{2}$), and female and male interaction ($\sigma_{fm}^{2}$) effects. ${\sigma_{gs}^{2}}^{*}$ is the variance component due to hybrids × environment interaction, which was computed as a summation of female × environment ($\sigma_{fs}^{2}$), male × environment ($\sigma_{ms}^{2}$), and female × male × environment ($\sigma_{fms}^{2}$). Likewise, ${\sigma_{a}^{2}}^{*}$ is additive genetic variance and is calculated as a summation of $\sigma_{f}^{2}$ and $\sigma_{m}^{2}$. ‘t’ and ‘r’ are the number of environments and replications within each environment, respectively. Coefficient of variation (CV_e_) was calculated using ${CV}_{e}=\;\frac{\sqrt{\sigma_{e}^{2}}}{\bar{x}}$, where $\sigma_{e}^{2}$ is residual variance and $\bar{x}$ is overall mean of the traits.

BLUEs were calculated for all phenotypic data by fitting fixed hybrid effects at each environment using Equation (1). Extracted BLUEs for hybrids were used to train prediction models. Kernel-based genomic best linear unbiased prediction (GBLUP) models were trained using SNP markers in silico for the genomic prediction (GP) model. The phenomic prediction (PP) model used cleaned NIR spectral data after filtering and pretreatment of the first derivative. Phenomic data were integrated with genomic data for the GP + PP model. All the statistical models were fitted for single and multi-environments and defined as single environment and combined environments.

### 5.7. Genomic and Phenomic Prediction Models

#### 5.7.1. Single-Environment Prediction Models

Three different predictive models were fitted to predict the phenotypic performance of the hybrids. These include genomic prediction (GP), phenomic prediction (PP), and genomic + phenomic prediction (GP + PP). GP was trained by modeling the GCA effects of females and males and SCA effects of female × male interactions using SNP markers. The first derivative of NIR was considered as a PP. Finally, phenomic data was integrated with genomic data for GP + PP models.

GP Model:

(4) $$y=1\mu +\;Z_{1}f+Z_{2}m+Z_{3}h+e$$

PP Model:

(5) $$y=1\mu +{NIR}_{1}+\;e$$

GP + PP Model:

(6) $$y=1\mu +{NIR}_{1}+{\;Z}_{1}f+Z_{2}m+Z_{3}h+e$$

where y is a vector of response variables; µ is intercept; f is a vector of GCA effects of females, f ~ N (0, σ^2^_fj_G_f_), where j is environments; m ~ N (0, σ^2^_mj_G_m_), m is a vector of GCA effects of males, h ~ N (0, σ^2^_hj_H), where h is a vector of SCA effects of hybrid combinations; e ~ N (0, σ^2^_ej_I), e is a vector of residuals. Notably, 1 is a vector of ones; Z_1_, Z_2_, and Z_3_ are incidence matrices for females, males, and hybrids, respectively. H is calculated by the Kronecker product in silico using G_f_ and G_m_. For phenomic kernels, cleaned NIR bands, i.e., the first derivative of NIR, were modeled like genomic markers; therefore, it follows the distribution of ${NIR}_{1}\sim^{i.i.d.}N\left(0,NIR\sigma_{p}^{2}\right)$, where $\sigma_{p}^{2}$ and NIR represent a phenomic variance component and phenomic effects estimated using the first derivative of NIR.

#### 5.7.2. Combined Environment Prediction Models

The single-environment models were extended to incorporate interactions with multi-environments. Environmental and G × E effects were modeled by including incidence matrices that relate phenotypic observation with the environment [52]. NIR × E interaction was computed using the Hadamard product since NIR was collected from grain samples of each environment. The Hadamard product (where ⨀ denotes the element-wise product) of the covariance structure of the interaction was modeled as follows: $f\times E\;\overset{i.i.d.\;}{\sim }\;N\left(0,\;\sigma_{f\times E\;}^{2}V_{f}\right)$, $m\times E\;\overset{i.i.d.}{\sim }\;N\left(0,\;\sigma_{m\times E\;}^{2}V_{m}\right)$ and $f\times m\times \;E\;\overset{i.i.d.}{\sim }\;N\left(0,\;\sigma_{H\times E\;}^{2}V_{H}\right)$; where $\sigma_{f\times E\;}^{2}$, $\sigma_{m\times E\;}^{2}$, and $\sigma_{H\times E\;}^{2}$ are variance components associated with female × environment, male × environment, and female × male × environment, respectively. V_F_, V_M_, and V_H_ represent variance–covariance matrices for female × environment, male × environment, and female × male × environments, respectively. Variance–covariance matrices were calculated as V_F_ = Z_1_G_f_Z’_1_⊙Z_E_Z’_E_, V_M_ = Z_2_G_m_Z’_2_⊙Z_E_Z’_E_, and V_H_ = Z_3_G_H_Z’_3_⊙Z_E_Z’_E_. For the interaction of phenomic effects, ${NIR}_{1}\times E\;\overset{i.i.d.\;}{\sim }\;N\left(0,\;\sigma_{P\times E\;}^{2}V_{P}\right)$ where $\sigma_{P\times E\;}^{2}$ are variance components associated with phenomic effects × environment and V_P_ = Z_P_P_H_Z’_P_⊙Z_E_Z’_E_.

GP Model:

(7) $$y=µ+Z_{1}f+Z_{2}m+Z_{3}h+Z_{4}E+Z_{5}fE+Z_{6}mE+Z_{7}fmE+\;e$$

PP Model:

(8) $$y=\mu +{NIR}_{1}+E+{NIR}_{1}E+\;e$$

GP + PP Model:

(9) $$y=µ+\;Z_{1}f+Z_{2}m+Z_{3}h+Z_{4}E+Z_{5}fE+Z_{6}mE+Z_{7}fmE+{NIR}_{1}+\;{NIR}_{1}E\;+\;e$$

#### 5.7.3. Cross-Validation Performance Evaluation

For the combined environment analysis, three different cross-validation (CV) scenarios were tested and repeated 50 times. These three scenarios represent the selection situations encountered in a breeding program. The CV1 method predicts hybrids that have not been evaluated in any environments; however, sets of genetically related hybrids have been evaluated in the same environment. The CV2 method (leave one environment out) predicts previously tested hybrids in uncharacterized environments. Likewise, the CV3 method predicts untested hybrids in uncharacterized environments (new lines in new environments). For CV1, 100 hybrids were partitioned into 70:30 (70 hybrids for training and 30 for testing purposes for all environments) (Supplementary Figure S3a). Training datasets of (70 × 8 = 560) were used to predict (30 × 8 = 240) records for agronomic traits: grain yield, days to anthesis, and plant height across eight environments. After fitting the models, Pearson’s correlation coefficients were calculated for testing sets of each environment for CV1. In a similar way, training datasets of (70 × 4 = 280) were used to predict testing sets of (30 × 4 = 120) for kernel characteristic traits from four environments.

For CV2 and CV3, phenotypic records for one of the environments were removed in addition to 70:30 partitions for CV1. In this case, the training set consists of (70 × 7 = 490) and testing sets of [(30 × 7) + 100 = 310] for agronomic traits (Supplementary Figure S3b). Similarly, training sets consist of (70 × 3 = 210) to predict testing sets comprising [100 + (30 × 3) = 190] for kernel characteristic traits. Pearson’s coefficient of correlation of test environments was computed for CV2 and CV3. In this analysis, 18CS and 19COL were chosen as uncharacterized environments for CV2 and CV3 schemes. Likewise, the models were fitted in a 70:30 ratio for a single environment without G × E kernels.

Pearson’s correlation coefficient on CV, model, and trait combinations was finally used to calculate mean and standard error for multi-environment models. Finally, post hoc analyses with Tukey’s honest test at the significance level of 0.05 were performed for prediction accuracies to detect differences between model performances for all traits.

### 5.8. Software

All models were fitted in R where SNP markers and NIR spectra were used for GP and PP models, respectively [53]. The first derivative of the matrix of NIR was calculated using the ‘SavitzkyGolay’ function from the library ‘prospectr’ to build phenomic relationship matrices [49]. All the prediction models were fitted with 5000 iterations, and burnIn of 1000 with thin of 10 using Gibbs sampler using the ‘BGLR’ package in R [54]. Genomic and phenomic priors were fitted as Reproducing Kernel Hilbert Spaces (“RKHS”); however, environment kernels were fitted using “BRR”. Three models, as explained above, were fitted for a single environment and multi-environment. G × E and NIR × E were calculated using Hadamard products.

## Figures and Tables

**Table 1:** Variance components and heritability estimates for agronomic and grain traits in 100 grain sorghum hybrids from a 10 × 10 complete factorial that were evaluated in eight Texas and Kansas multi-locations in summer 2018 and 2019.

Variance Components ^a^	Agronomic Traits						Kernel Traits					
	Grain Yield (GY)		Days to Anthesis (DA)		Plant Height (PH)		Kernel Hardness INDEX (KHI)		Kernel Diameter (KD)		Kernel Weight (KW)	
	Estimate	%	Estimate	%	Estimate	%	Estimate	%	Estimate	%	Estimate	%
(Hybrid)	0.39	14.5	6.23	11.8	94.6	48.1	76.6	56.7	0.023	54.1	4.76	41.5
GCA_f_	0.18 ***	6.8	3.03 ***	5.7	35.1 ***	17.8	18.6 ***	13.8	0.009 ***	20.8	1.51 ***	13.2
GCA_m_	0.18 ***	6.6	2.93 ***	5.5	51.0 ***	25.9	42.8 ***	31.7	0.013 ***	29.3	2.34 ***	20.5
SCA	0.03 **	1.2	0.28 ***	0.5	8.5 ***	4.3	15.2 ***	11.2	0.001 ***	4.05	0.90 ***	7.85
Env	1.30 ***	48.6	41.20 ***	78.0	51.0 ***	25.9	11.1 ^NS^	8.2	0.01 **	23.1	2.45 **	21.4
(Hybrid × Env)	0.32	12.1	2.58	4.9	15.2	7.7	58.2	19.4	0.003	10.8	1.76	15.4
GCA_f_ × Env	0.16 ***	6.0	1.41 ***	2.7	4.1 ***	2.1	9.3 ***	6.9	0.001 ***	3.65	0.57 ***	4.94
GCA_m_ × Env	0.09 ***	3.5	1.03 ***	1.9	8.5 ***	4.3	10.8 ***	8.0	0.001 ***	3.21	0.50 ***	4.39
SCA × Env	0.078 *	2.6	0.14 ^NS^	0.3	2.7 *	1.4	6.1 ***	4.5	0.001 ***	3.93	0.69 ***	6.05
Rep (Env)	0.02 ^NS^	0.9	0.14 ^NS^	0.3	1.3 ^NS^	0.6	2.3 ^NS^	1.6	0 ^NS^	1.54	0.170 ^NS^	1.48
Residual	0.634	23.8	2.65	5.0	34.6	17.6	18.7	13.8	0.004	10.5	2.31	20.2
*H* ^2^	0.83		0.92		0.96		0.90		0.93		0.87	
*h* ^2^	0.76		0.88		0.87		0.72		0.86		0.70	
*h* ^2^ *_f_*	0.38		0.44		0.35		0.22		0.36		0.27	
*h* ^2^ *_m_*	0.38		0.45		0.52		0.50		0.50		0.43	
CVe	12.50		2.31		4.36		5.76		2.75		5.88	

^a^ GCA_f_, female general combining ability; GCA_m_, male general combining ability; SCA, specific combining ability. ^NS^ Non-significant. * significant at the 0.05 probability level. ** significant at the 0.01 probability level. *** significant at the 0.001 probability level. *H*^2^: Broad sense heritability, *h*^2^: narrow sense heritability; *h*^2^*_f_*: female narrow sense heritability; *h*^2^*_m_*: male narrow sense heritability; CVe: coefficient of variation.

## Data Availability

Sapkota, P. (2025). Supplementary data—phenomic and genomic models for predicting sorghum agronomic and grain characteristics related traits across environments. https://github.com/sapkotapradip/Phenomic_NIR-Genomic_Pub (accessed on 27 July 2025).

## Supplementary Materials

The following supporting information can be downloaded at: https://www.mdpi.com/article/10.3390/plants14182871/s1, Figure S1: Percent variation explained by male effects, female effects, female × male effects, and replication across all 4200 near-infrared spectroscopy (NIRS) bands across eight environments by equation $y=1µ+Z_{1}f+Z_{2}m+Z_{3}h+\;e$. Abbreviations for locations are COL: Colby, KS, CS: College Station, TX, GC: Garden City, KS, VC: Victoria, TX, TA: Taft, TX. Figure S2: Prediction abilities of Genomic Prediction (GP), Phenomic Prediction (PP), GP + PP models in predicting GY: Grain Yield, PH: Plant Height, DA: Days to Anthesis, KHI: Kernel Hardness Index, KD: Kernel Diameter, and KW: Kernel Weight for eight environments (18COL: 2018 Colby, KS; 18CS: 2018 College Station, TX; 18GC: 2018 Garden City, KS; 18VC: 2018 Victoria, TX; 19COL: 2018 Colby, Kansas; 19CS: 2019 College Station, TX; 19TA: 2019 Taft, TX; 19VC: 2019 Victoria, TX). Bars represent standard deviation for each model within environments for respective traits. Figure S3: Cross validation scheme across environments for genomic and phenomic models in predicting sorghum hybrid performance. (a) CV1 cross validation scheme across environments with 30% testing hybrids i.e., 30 × 8 = 240, which were predicted by training 70% of hybrids across environments i.e., 70 × 8 = 560. (b) CV2 and CV3 cross validation schemes were performed by leaving one environment out on top of 30% of hybrids from other seven environments, prediction accuracy was calculated for 70% tested hybrids on uncharacterized environments for CV2 and 30% untested hybrids in uncharacterized environments for CV3 as shown above. Prediction accuracy was calculated by calculating Pearson’s correlation coefficients within testing sets for all CV schemes.

## Abbreviations

The following abbreviations are used in this manuscript:

GS	Genomic selection
NIRS	Near-infrared spectroscopy
PS	Phenomic selection
KHI	Kernel hardness index
GY	Grain yield
DA	Days to anthesis
PH	Plant height
KD	Kernel diameter
KW	Kernel weight
SKCS	Single-kernel characteristics system
BLUE	Best linear unbiased estimates
LRT	Likelihood ratio test
GCA	General combining ability
SCA	Specific combining ability
G × E	Genotype × environment
H^2^	Broad sense heritability
h^2^	Narrow sense heritability
GP	Genomic prediction
PP	Phenomic prediction
