# Supplementary material for: Near-Infrared Spectroscopy-Based Phenomics Data Can Improve Genomic Prediction of Agronomic and Grain Quality Traits Across Multi-Environment Sorghum Hybrid Trials
Source: Plants (Basel). 2025 Sep 15;14(18):2871. doi: 10.3390/plants14182871 (PMC12473895; doi:10.3390/plants14182871)
Supplement: Supplementary file 1 [file plants-14-02871-s001.zip › plants-3822776-supplementary.pdf]

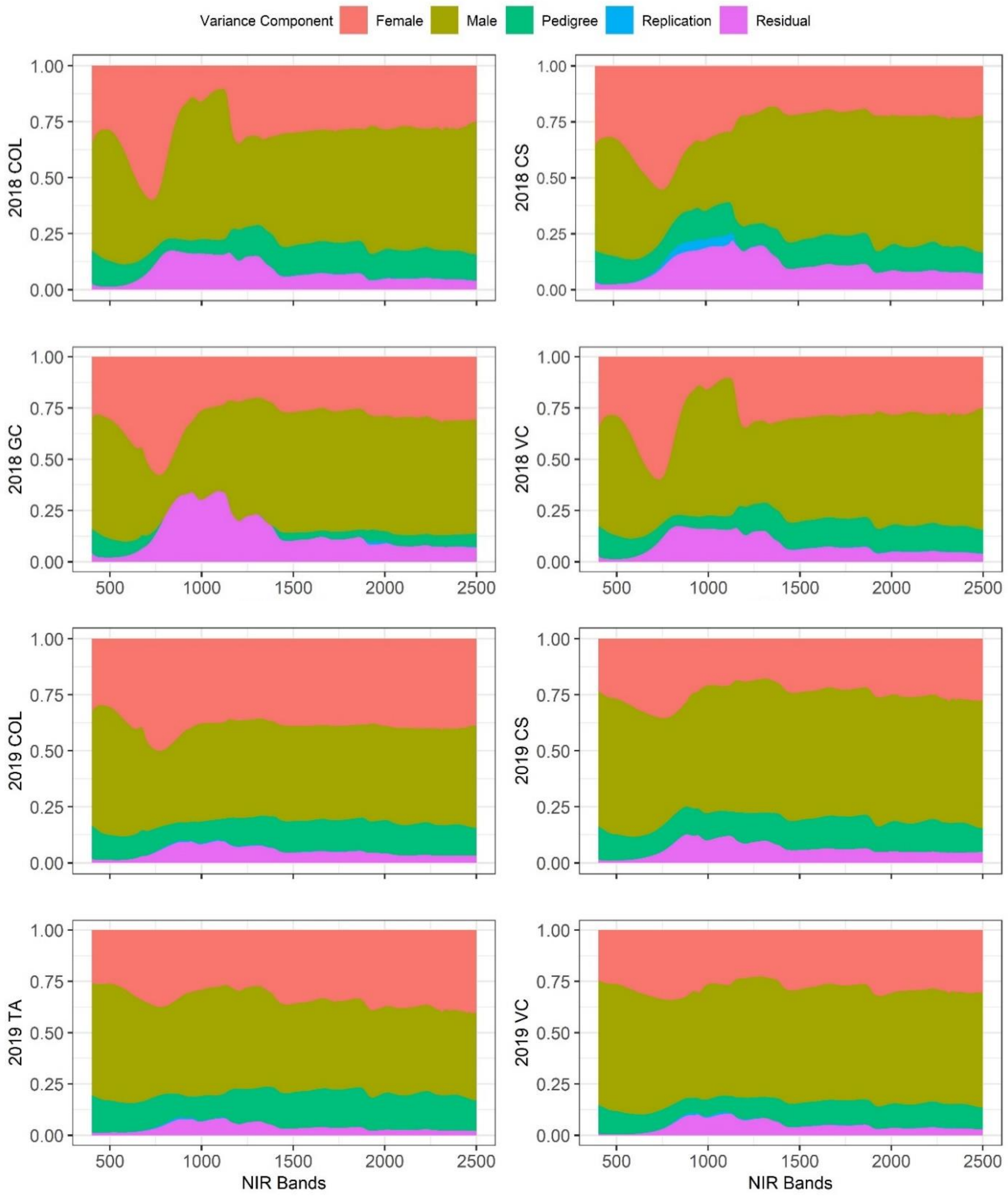

Supplementary Figure S1: Percent variation explained by male effects, female effects, female × male effects, and replication across all 4200 near-infrared spectroscopy (NIRS) bands across eight environments by equation $y = 1\mu + Z_1 f + Z_2 m + Z_3 h + e$. Abbreviations for locations are COL: Colby, KS, CS: College Station, TX, GC: Garden City, KS, VC: Victoria, TX, TA: Taft, TX.

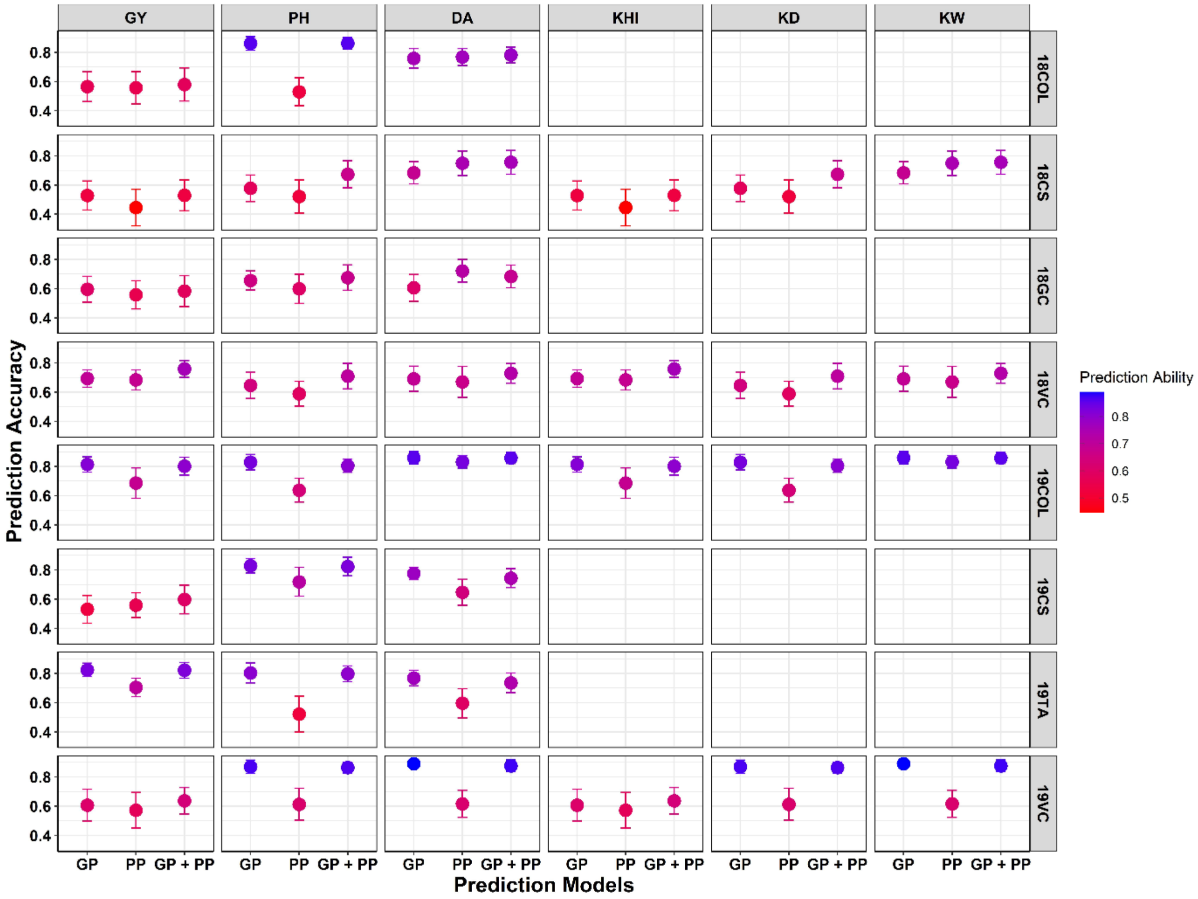

Supplementary Figure S2: Prediction abilities of Genomic Prediction (GP), Phenomic Prediction (PP), GP + PP models in predicting GY: Grain Yield, PH: Plant Height, DA: Days to Anthesis, KHI: Kernel Hardness Index, KD: Kernel Diameter, and KW: Kernel Weight for eight environments (18COL: 2018 Colby, KS; 18CS: 2018 College Station, TX; 18GC: 2018 Garden City, KS; 18VC: 2018 Victoria, TX; 19COL: 2018 Colby, Kansas; 19CS: 2019 College Station, TX; 19TA: 2019 Taft, TX; 19VC: 2019 Victoria, TX). Bars represent standard deviation for each model within environments for respective traits.

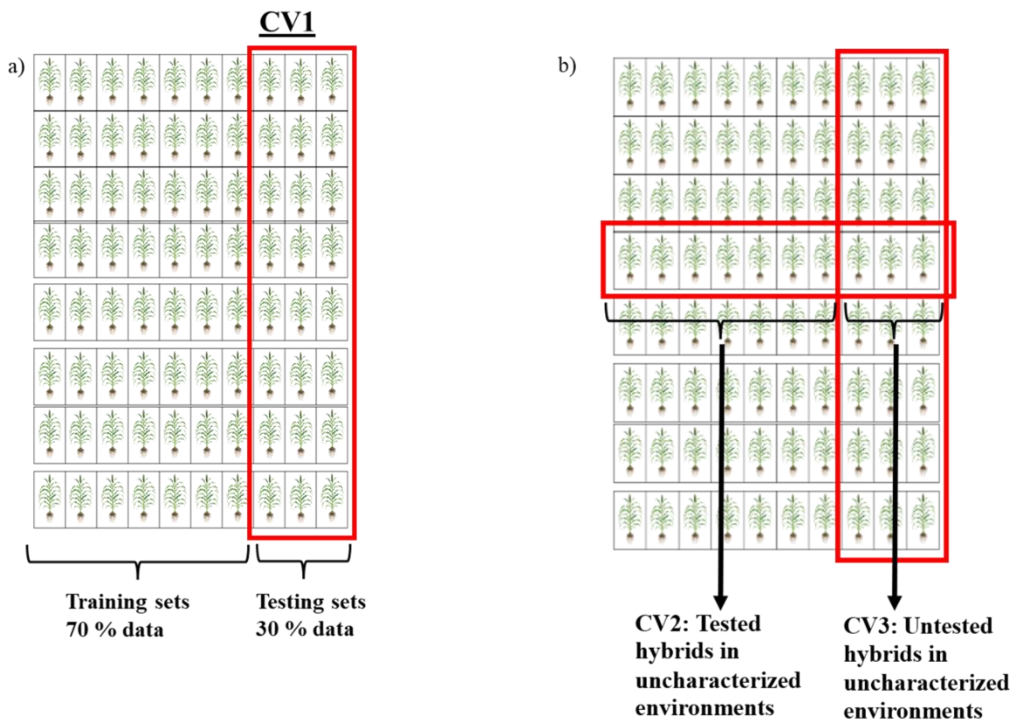

Supplementary Figure S3: Cross validation scheme across environments for genomic and phenomic models in predicting sorghum hybrid performance. a) CV1 cross validation scheme across environments with 30% testing hybrids i.e. 30 × 8 = 240, which were predicted by training 70 % of hybrids across environments i.e. 70 × 8 = 560. b) CV2 and CV3 cross validation schemes were performed by leaving one environment out on top of 30 % of hybrids from other seven environments, prediction accuracy was calculated for 70% tested hybrids on uncharacterized environments for CV2 and 30 % untested hybrids in uncharacterized environments for CV3 as shown above. Prediction accuracy was calculated by calculating Pearson's correlation coefficients within testing sets for all CV schemes.
